# Clinical and pathological analysis of the kidney in patients with hypertensive nephropathy

**DOI:** 10.3892/etm.2013.1306

**Published:** 2013-09-18

**Authors:** XIANG-CHUAN WANG, CHUN-HUI LIU, YUN-JING CHEN, YANG WU, LEI-SHAN YANG, HONG-MIN LIU, HAI-LI LIAO

**Affiliations:** Department of Nephrology, No. 163 Hospital of PLA, Changsha, Hunan 410003, P.R. China

**Keywords:** hypertensive, nephropathy, kidney, pathology

## Abstract

The aim of the present study was to identify the association between pathological types of kidney and clinical manifestations in patients with hypertensive nephropathy. The blood pressure, fundus, urinalysis test results and renal function changes were analysed in patients who were treated for hypertensive nephropathy. Downward kidney puncture biopsy was performed using a 16G ejection needle with the aid of B ultrasound in 47 cases. The specimens were observed using light microscopy and immunofluorescence. The pathological changes observed in the patients exhibiting symptoms of hypertensive nephropathy varied. The majority of clinical manifestations were benign arteriolar nephrosclerosis, hyaline degeneration of the renal artery and the appearance of a thickened wall of a thickened renal artery wall. Severe cases showed malignant arteriolar nephrosclerosis characterised by fibrinoid necrosis of renal arterioles and intimal hyperplasia. In addition, in the severe cases, fibrinoid necrosis of the afferent arteriole and arcuate artery wall was observed, with severe interlobular artery and arcuate artery myointimal thickening. Renal biopsy in patients with hypertensive nephropathy is safe and feasible. The prognosis and treatment of pathological and clinical disease related to renal pathology is necessary.

## Introduction

The rate of hypertension caused by end-stage renal disease is continuously increasing. Hypertensive renal disease has become the third most common cause of chronic renal failure among patients subjected to dialysis. Among the elderly, chronic renal insufficiency is the most common cause of hypertensive renal disease (1). As hypertensive nephropathy in patients with early renal damage often occurs without symptoms (2), these patients do not undergo medical check up. Once the clinical manifestations or routine testing results have been identified to be abnormal, a considerable number of renal lesions may have already developed (3). Thus, the prevention and early diagnosis of hypertensive nephropathy is necessary. Renal needle biopsy is one of the most effective methods for determining the degree of kidney damage. Active antihypertensive and other forms of treatment have been administered to interrupt the vicious cycle of kidney disease and high blood pressure (4). Damage to organs, such as the kidneys, as a result of high blood pressure is potentially reduced. In the present study, a total of 47 patients with hypertensive nephropathy who underwent renal biopsy were analysed with the aim of investigating the association between pathological types of kidney and clinical manifestations.

## Materials and methods

### Patients

From July 2008 to October 2010, 47 patients (33 males and 14 females) aged 38–65 years (average age, 42.8 years) were included in the present study. The longest and shortest durations of hypertension were 35 and 6 years, respectively (average, 15.7 years). The present study was conducted in accordance with the Declaration of Helsinki and approved by the Ethics Committee of No. 163 Hospital of PLA (Changsha, China). Written informed consent was obtained from all the participants.

### Diagnostic criteria

The criteria for the diagnosis of hypertensive nephropathy (5) were as follows: i) primary hypertension; ii) >5 years of sustained hypertension before proteinuria; iii) persistent proteinuria (generally mild to moderate) with less visible components detected by microscopic examination; iv) retinal arteriosclerosis or arteriosclerotic changes in the retina; v) various primary renal diseases were excluded; and vi) other secondary renal diseases were also excluded. A history of hypertensive left ventricular hypertrophy, coronary heart disease, heart failure, cerebral arteriosclerosis and/or history of cerebral vascular accident, hyperuricemia, renal tubular dysfunction preceding renal function damage, slow progression and other factors were used as auxiliary diagnostic conditions.

### Clinical analysis

The patients underwent the following laboratory tests: routine blood and urine examinations; 24-h urinary protein excretion measurements; plasma total protein and albumin, serum creatinine (Scr) and blood urea nitrogen tests; serum IgG, IgA, IgM, C3 and C4 detection; and plasma renin level monitoring. All the patients underwent fundus examination based on the standard classification of fundus lesions according to the Keith-Wagener classification system (6) with the following grades: I, vascular systolic; II, exudative phase; III, hardening period; and IV, complicated stage. B ultrasound and chest X-ray examination of the kidneys were also conducted. Computed tomography (CT) of the head was conducted when necessary to determine the damage of the target organ caused by hypertension.

### Histological examination

The administration of intravenous antihypertensive agents was discontinued. Renal puncture biopsy was then conducted using a 16G ejection needle with the aid of B ultrasound. Renal biopsy specimens were examined under a light microscope with the following methods: haematoxylin and eosin (H&E), periodic Schiff-methenamine silver (PASM) and Masson’s staining; immunofluorescence for IgG; as well as IgA, IgM, C3, C4 and C1q examination.

### Treatment

Based on the cardiac and renal functions of the patients, drug combination therapies with two or more antihypertensive agents such as calcium channel blockers (CCBs) (7), angiotensin-converting enzyme inhibitors (ACEIs)/angiotensin II-1 receptor antagonist (ARB)s, β-receptor blockers, α-blockers and diuretics were selected. An intravenous infusion of sodium nitroprusside was administered if a hypertensive crisis was observed at the beginning of the course. The blood pressure decreased to 160–170/100–110 mmHg within 24 h from beginning of the course. The administration of intravenous antihypertensive drugs was gradually discontinued and oral antihypertensive therapy was provided until the blood pressure became stable.

### Statistical analysis

The data were expressed as the mean ± standard deviation. Statistical analysis was conducted by group t-test using SPSS 12.0 software (SPSS, Inc., Chicago, IL, USA). Data counting was conducted using the χ^2^ test. P<0.05 was considered to indicate a statistically significant result.

## Results

### General information

Changes in blood pressure and the fundus were observed among the 47 patients who had not received regular antihypertensive treatment outside the hospital. The blood pressure levels monitored at the hospital reached 150–280/105–190 mmHg. The average blood pressure was 185±33/102±30 mmHg. The fundus changes that were observed were as follows: five cases of grade I; 13 cases of grade II; 24 cases of grade III; and five cases of grade IV.

Among the 47 patients with various degrees of proteinuria, 13 patients had a 24-h urinary protein excretion of <1 g, 24 had a 24-h urinary protein of 1–3.5 g and 10 had a 24-h urinary protein excretion of >3.5 g. Furthermore, 26 patients had microscopic haematuria, none of the patients had gross haematuria, 35 had normal renal function, 10 had mild renal impairment (Scr, 133–422 *μ*mol/l) and two had severe renal function impairment (Scr >422 *μ*mol/l). A longer duration of hypertension and higher 24-h urinary protein excretion levels indicated more advanced kidney damage. When the patients were grouped according to the duration of the disease, there were significant differences among groups with different duration of disease (P<0.05; Table I).

### Target organ symptoms

Among the total number of patients, 47 (100%) had headaches, 19 (40.42%) had blurred vision, 32 (68.09%) had left ventricular hypertrophy, six (12.77%) had left heart failure and two (4.26%) had anaemia. The clotting function was normal in all cases.

### Pathological changes in the kidney

The patients (n=47) who underwent renal biopsy showed changes indicative of varying degrees of microscopic or original haematuria (which was reduced or absent within 48 h). No gross haematuria occurred, and no additional complications were observed. Pathological examinations showed that the renal artery, renal parenchyma and renal interstitial cells had varying degrees of damage. The urinary protein level was <1 g, and no microscopic haematuria was observed. In the majority of the cases, the renal function was normal or modestly impaired, and the patients showed benign nephrosclerosis of small arteries. In 41 cases, the following conditions were observed under a light microscope (PASM staining; magnification, ×100): afferent renal arteriolar hyalinisation, interlobular arteries and arcuate artery myointimal thickening, glomerular capillary plexus collapse, and basement membrane ischaemic shrinkage. The following observations were made after H&E staining under a light microscope (magnification, ×100): tubular atrophy, regeneration, and renal interstitial oedema. An increased number of cases of proteinuria and haematuria was observed in patients with severe renal disease along with malignant arteriolar nephrosclerosis. The following conditions were observed under a light microscope (PASM staining; magnification, ×100) in six cases: fibrinoid necrosis of the afferent arteriole and arcuate artery wall, severe interlobular artery and arcuate artery myointimal thickening, and glomerular segmental fibrinoid necrosis. Tubular acute diffuse injury and renal parenchymal fibrosis were observed following H&E staining under a light microscope (magnification, ×100). The immunofluorescence results were as follows: various degrees of immune complex deposition were visible in the glomerular basement membrane; IgM was positive in 20 patients (+ or ++). C3-positive results were observed in 12 cases (±-++). Multiple depositions of immune complexes were observed in several cases. A longer duration of hypertension correlated with higher levels of immune complex deposition (P<0.05; Table II).

### Curative effect

Following treatment, the blood pressure of all 47 patients returned to normal. The 24-h urinary protein excretion in 41 cases was <150 mg. In 10 cases, the renal function was modestly impaired. In eight cases, Scr returned to normal. In two cases, renal function was severely impaired. The blood creatinine level of one patient was 362.31 *μ*mol/l. In another patient, the blood creatinine level continued to increase, reaching 743.62 *μ*mol/l until blood dialysis was conducted.

## Discussion

Hypertensive nephropathy in patients with pathological renal changes may occur in hypertensive patients who have suffered from this disease for 5–10 years (8). This finding has been mainly demonstrated in vascular disease, ischaemic glomerular changes and acute tubular interstitial injury (9). The degree of kidney damage increases with the duration of extended hypertension. Pathological examination has shown that this disease mainly affects the small renal arteries of the glomerulus (10), causing afferent arteriolar hyalinisation, interlobular thickening of the artery and arcuate artery endomysium, as well as subsequent manifestations secondary to renal parenchymal damage (10). Hypertensive renal damage caused by renal ischaemia and glomerular ischaemia initially occurs when the capillary plexus collapses and the basement membrane shrinks; glomerular segmental sclerosis occurs until ball sclerosis is observed. Tubular ischaemia causes tubular atrophy, basement membrane thickening, renal mesenchymal focal mononuclear cell infiltration and fibrosis. Numerous remnants of healthy kidney cells indicate glomerular and tubular compensatory hypertrophies when glomerulosclerosis and tubular atrophy simultaneously occur in the ischaemic kidney. Hypertrophy of the remaining kidney nephrons affected by ischaemic atrophy is significantly different; the glomeruli is in a state of high pressure, high perfusion and high filtration (11). This state influences a compensatory mechanism that allows residual renal units to compensate a complete renal excretory function. However, the ‘three high’ haemodynamic changes possibly promote glomerulosclerosis, resulting in no ischaemic changes in the nephrons affected by the compensatory hypertrophy that occurs in glomerular and tubular atrophies.

The clinical symptoms of hypertensive kidney damage are often observed when hypertension is persistent for 10–15 years (4). The earliest clinical manifestations are increased nocturia and induced onset of proteinuria (12). As hypertension is exacerbated, patients with impaired renal function gradually exhibit increased Scr until chronic renal failure occurs (13). Among the elderly, the coexistence of diabetes, hyperlipidaemia and hyperuricaemia in patients manifesting these clinical symptoms may occur earlier (14,15). Given that the clinical symptoms of hypertensive nephropathy appear later than the pathological changes, hypertensive nephropathy is usually ignored (16) and is not identified in general routine examinations. Although a medical doctor may notice the possibility of hypertensive renal damage, he or she may be satisfied with only conventional test results. Consequently, hypertensive nephropathy in patients with asymptomatic and negative laboratory test results is not taken seriously (15). Thus, the opportunity for early diagnosis and treatment is lost.

Considering that hypertension aggravates renal dysfunction and that renal dysfunction stimulates blood pressure, kidney function is associated with blood pressure (15). Patients with long-term benign hypertension mainly have benign nephrosclerosis in small arteries, autoregulation of the renal artery, and normal glomerular fibrinoid necrotic lesions. Once renal dysfunction occurs, the renin-angiotensin-aldosterone system (RAS system) becomes activated (17), causing a significant increase in blood pressure and changes in malignant hypertension (18). Such patients often experience increases in renin and catecholamine levels, leading to a vicious cycle with the development of malignant renal sclerosis of the small arteries and abnormal autoregulation of the renal artery. These consequences are often accompanied by glomerular fibrinoid necrosis. Renal needle biopsy is one of the most effective methods used to determine the degree of kidney damage. To distinguish between benign and malignant renal arteriosclerosis, the early identification of hypertension-induced renal dysfunction is a basis for the selection of reasonable treatment options. Active antihypertensive and other treatments may interrupt the vicious cycle of effects between the kidney and high blood pressure as well as reduce the damage caused by high blood pressure on target organs such as the kidneys.

In the present study, 47 cases of renal biopsy with no serious complications were examined. The blood pressure of all patients returned to normal. In addition, 41 cases showed a 24-h urinary protein ratio of <150 mg, 10 patients had mild renal impairment and eight patients had a Scr that returned to normal. Only one case of severe renal impairment was observed, and the renal function of this patient was not effectively improved following treatment. When the treatment is timely and appropriate measures are taken to positively control the blood pressure (19), the prognosis is usually good and the impaired renal function recovers to various extents (20). Thus, renal biopsy is important for identifying the causes of hypertensive nephropathy. This procedure also provided basic treatment information in a safe and feasible manner.

## Figures and Tables

**Table I.** Association between the duration of hypertension and hypertensive renal disease (n).

Duration of disease (years)	n	Microscopic haematuria	Proteinuria (g/24-h urine)			Scr level (*μ*mol/l)		
			<1 g	1–3 g	>3 g	Normal	133–422	>422
6–10	15	2	9	6	0	14	1	0
11–15	12	7	3	7	2	9	3	0
16–20	14	11	1	10	3	10	4	0
>20	6	6	0	1	5	2	2	2
Total	47	26	13	24	10	35	10	2

Scr, serum creatinine.

**Table II.** Association between the duration of hypertension and kidney tissue immunofluorescence findings.

Duration of disease (years)	n	Immunofluorescence results (positive, n)					
		IgG	IgA	IgM	C3	C4	C1q
6–10	15	0	1	3	1	0	0
11–15	12	0	2	4	2	0	0
16–20	14	1	4	8	5	1	1
>20	6	0	2	5	4	0	1
